# Supplementary material for: PVDF/KNO3 Composite Sub-Microfibers Produced by Solution Blow Spinning as a Hydrophobic Matrix for Fertilizer Delivery System
Source: Polymers (Basel). 2022 Mar 1;14(5):1000. doi: 10.3390/polym14051000 (PMC8912621; doi:10.3390/polym14051000)
Supplement: Supplementary file 1 [file polymers-14-01000-s001.zip › polymers-1596584-supplementary.pdf]

Supplementary material

PVDF/KNO_3 composite sub-microfibers produced by solution blow spinning as a hydrophobic matrix for fertilizer delivery system

Fabio Sobral[1], Michael J. Silva[2], Thalita Canassa[1], Além-Mar Goncalves[1,3], Cícero Cena[3]*

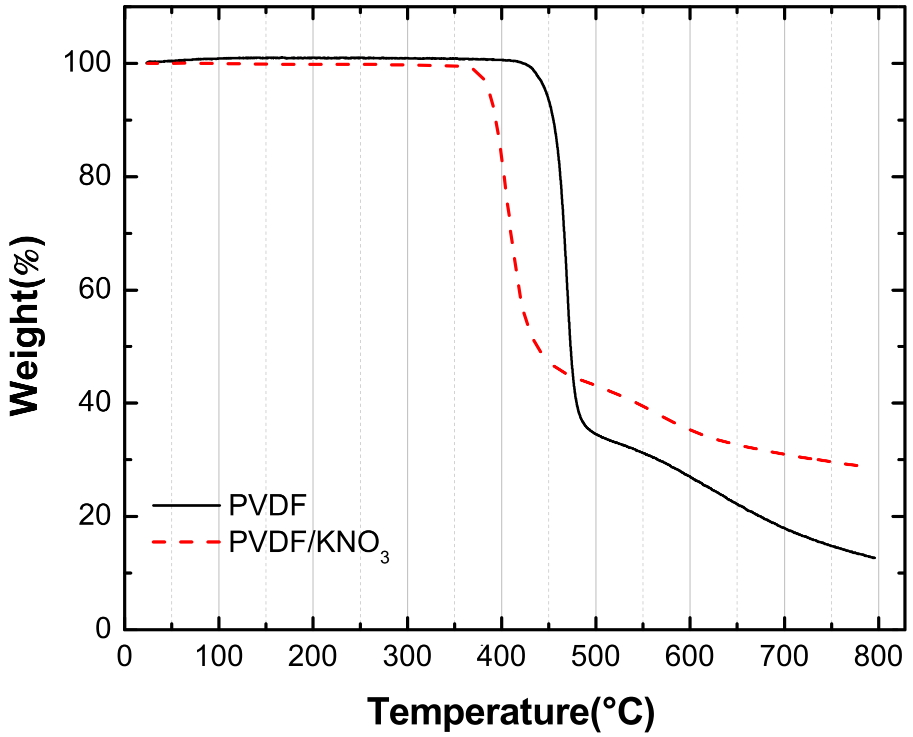

Figure S1: Thermogravimetric analysis (TGA), under nitrogen atmosphere with 60 ml/min steady flow, from room temperature up to 800 °C to evaluate the thermal stability of the samples.
